# Heart transplantation for rapidly progressive dilated cardiomyopathy complicated by an inferolateral left ventricular aneurysm: a case report

**DOI:** 10.3389/fcvm.2026.1789290

**Published:** 2026-10-26

**Authors:** Zhihua Yu, Li Yang, Ye Xu, Zuomin Li, Lijun Wang, Liqun He

**Affiliations:** 1 Department of Cardiology, Wuhan No. 1 Hospital, Wuhan, Hubei, China; 2 Department of Cardiothoracic Surgery, The Affiliated Tianyou Hospital of Wuhan University of Science and Technology, Wuhan, Hubei, China

**Keywords:** coronary artery disease, dilated cardiomyopathy, heart transplantation, left ventricular aneurysm, mitral regurgitation, myocardial infarction

## Abstract

**Background:**

Left ventricular (LV) aneurysm most commonly follows transmural myocardial infarction and is rarely described in dilated cardiomyopathy (DCM). When DCM and ischemic injury coexist, the presentation may be atypical and progression can be rapid.

**Case summary:**

A 52-year-old man developed chest tightness and dyspnea after an upper respiratory tract infection. Echocardiography showed LV dilatation with reduced systolic function (LVEF 36%). Coronary angiography on hospital day 4 demonstrated severe stenoses of the left circumflex/obtuse marginal branch and a moderate right coronary artery lesion, and a drug-eluting stent was implanted in the left circumflex artery. One month later, he was readmitted with recurrent dyspnea, BNP 2,627 ng/L, complete left bundle branch block, marked LV dilatation (end-diastolic diameter 113 mm), severe functional mitral regurgitation, and a large inferolateral apical LV aneurysm (74 mm × 50 mm) with paradoxical motion. Despite intensified heart failure therapy, he progressed to end-stage heart failure and underwent heart transplantation. Histopathology of tissue adjacent to the aneurysm was consistent with myocardial infarction with chronic inflammatory cell infiltration, and the sampled left circumflex artery showed atherosclerotic stenosis.

**Conclusion:**

In DCM with new regional wall motion abnormalities or rapid structural deterioration, concomitant ischemic injury should be considered even when symptoms are non-specific. Electrical and mechanical dyssynchrony may increase regional wall stress and contribute to aneurysm formation, accelerating clinical decline.

## Introduction

Left ventricular aneurysm is typically a consequence of transmural myocardial infarction and is less commonly associated with other conditions such as hypertrophic cardiomyopathy, cardiac sarcoidosis, Chagas disease, myocarditis, congenital LV aneurysm/diverticulum, or post-traumatic pseudoaneurysm (1–6). In idiopathic DCM, LV aneurysm has been reported but remains uncommon, and proposed mechanisms include regional fibrosis, intraventricular conduction disturbance, and heterogeneous wall stress. We report a case of newly diagnosed DCM with rapid LV enlargement complicated by an inferolateral apical LV aneurysm linked to ischemic injury, ultimately requiring heart transplantation.

## Case presentation

A 52-year-old man was admitted with a 5-day history of chest tightness and shortness of breath that began after a common cold. Symptoms were worse at night and with exertion. He reported cough with sputum but no fever and denied orthopnea. His medical history was notable for type 2 diabetes mellitus treated long-term with metformin. He denied known hypertension, dyslipidemia, or prior coronary artery disease. He had smoked approximately 20 cigarettes per day for more than 30 years and did not drink alcohol. There was no remarkable family history. On arrival, temperature was 36.5 °C, heart rate 78 bpm, respiratory rate 22/min, and blood pressure 130/70 mmHg. He was alert. Jugular venous distension was present. Lung auscultation revealed no rales. Cardiac examination showed leftward displacement of the cardiac border with a regular rhythm and no audible murmurs. No lower-extremity edema was noted. Initial laboratory testing demonstrated leukocytosis (11.59 × 10^9^/L) and thrombocytosis (419 × 10^9^/L) with a neutrophil count of 8.72 × 10^9^/L. High-sensitivity C-reactive protein was 25.75 mg/L and fasting glucose was 18.98 mmol/L. Lipids were: total cholesterol 3.49 mmol/L, HDL-C 0.84 mmol/L, LDL-C 2.27 mmol/L, and triglycerides 1.16 mmol/L. Cardiac enzymes, liver and renal function tests, antiviral myocarditis antibodies, and cytokines were within normal ranges. Electrocardiography showed sinus tachycardia (131 bpm), intraventricular conduction delay (QRS 128 ms), and poor R-wave progression. Transthoracic echocardiography demonstrated left atrial enlargement (anteroposterior diameter 50 mm), LV dilatation (LV end-diastolic diameter 6.37 cm), interventricular septal thickness 9 mm, and reduced systolic function (LVEF 36%) (Figure 1). Segmental dysfunction was noted, with reduced motion and systolic thickening of the LV posterior wall at the papillary muscle level. Given chest tightness with poor R-wave progression and regional wall motion abnormality, coronary angiography was performed on hospital day 4. The left main and left anterior descending arteries had no significant stenosis and the blood flow was TIMI 3. The LCX showed diffuse severe stenosis (approximately 80%) distal to the origin of the first obtuse marginal branch (OM1) with TIMI 2 flow, and the OM1 ostium had a focal severe stenosis (approximately 90%). The mid right coronary artery had a focal moderate-to-severe stenosis (approximately 70%) with TIMI 3 flow. These findings were concordant with the echocardiographic posterior wall hypokinesis. A drug-eluting stent (3.5 mm × 24 mm) was implanted from the proximal to distal LCX with a satisfactory angiographic result (Figure 2).

**Figure 1:**
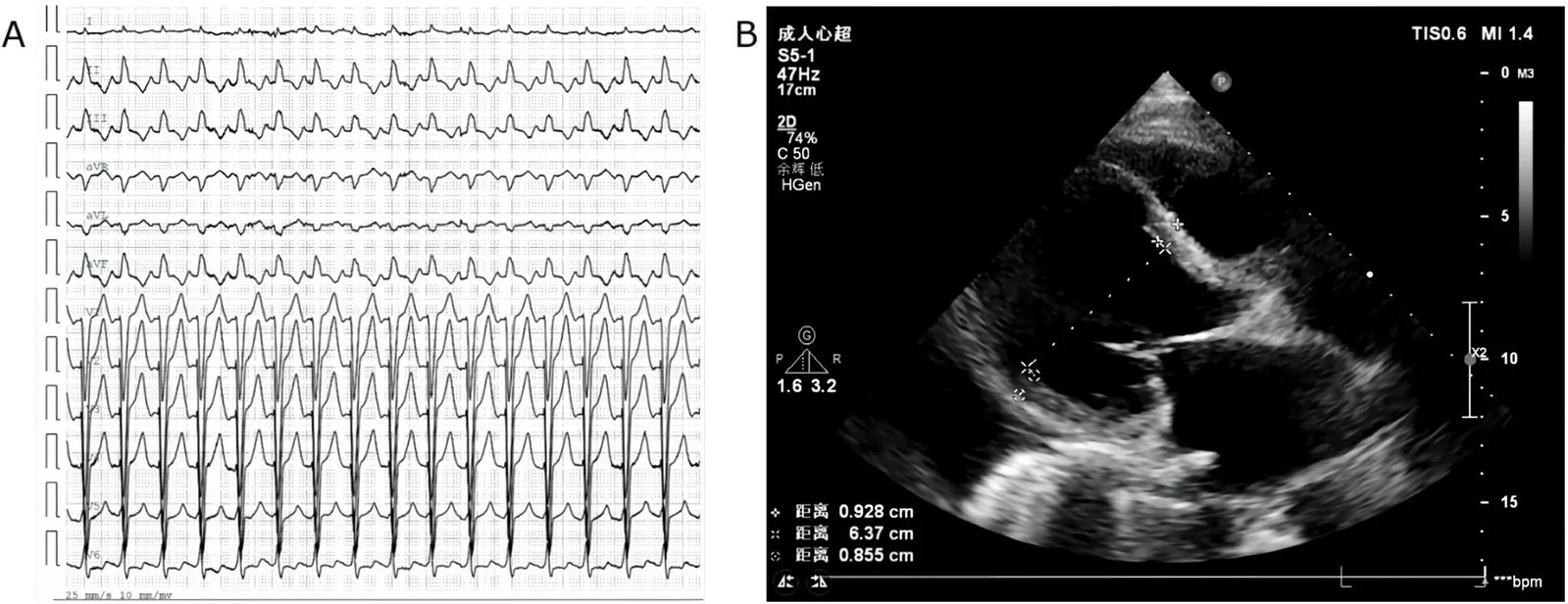
**(A)** Electrocardiogram on admission. Atrial rhythm, heart rate 131 bpm, QRS duration 128 ms, QTc interval 501 ms. **(B)** Transthoracic echocardiography on admission. Long-axis view of left ventricle showing LVIDd = 6.37 cm. LVIDd, left ventricular internal dimensions in diastole.

**Figure 2:**
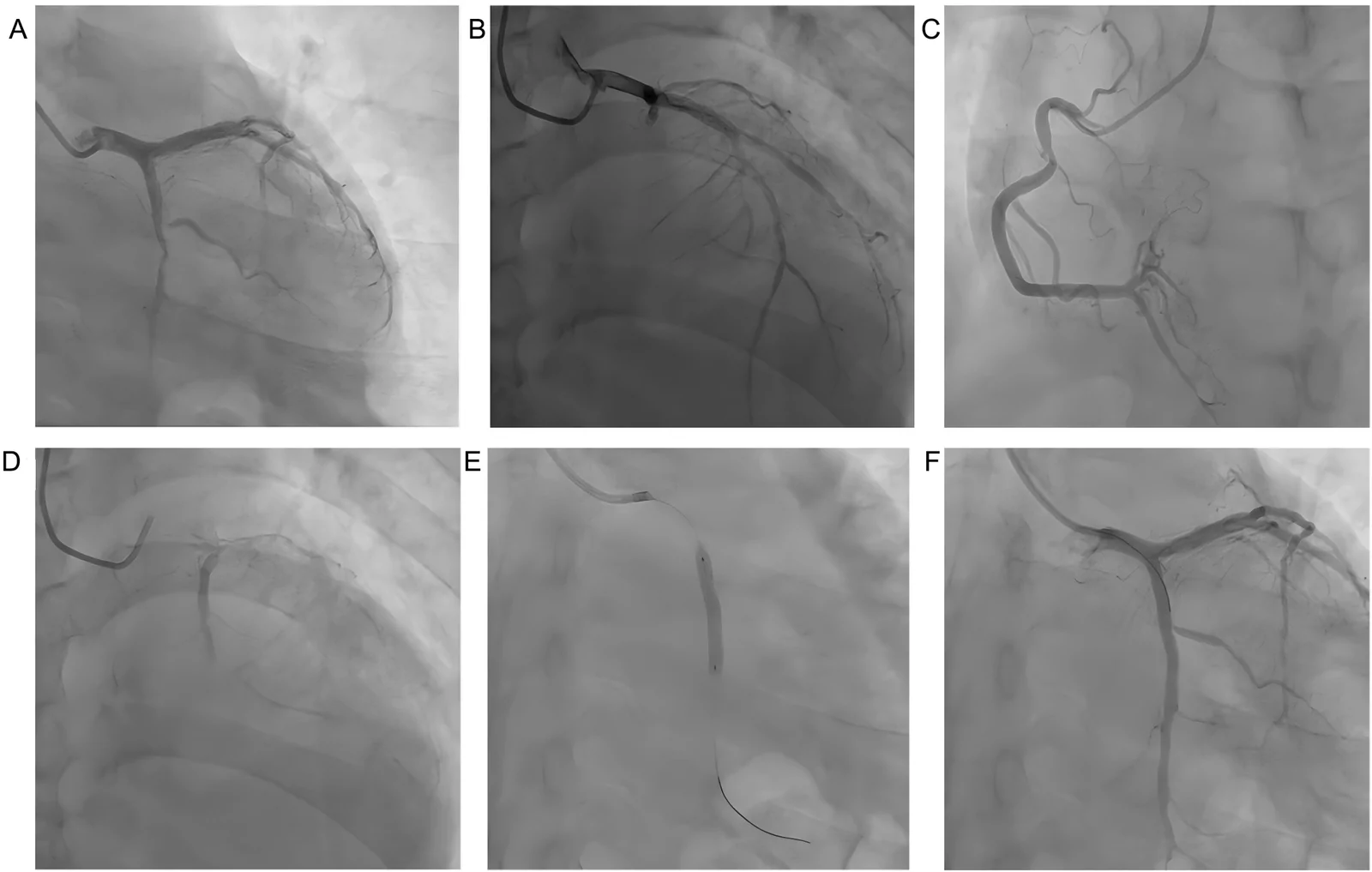
Coronary angiography. **(A)** Liver view showing severe stenosis at the LCX ostium after the OM1 branch origin, accompanied by distal TIMI 2 flow. **(B)** Right anterior oblique cranial view showing no significant stenosis lesion in LAD. **(C)** Left anterior oblique cranial view showing A 70% stenosis lesion observed in the mid-segment of the RCA with TIMI grade 3 flow. **(D)** LCX distal delayed filling. **(E)** 3.5 mm × 24 mm Drug-coated stent releasing. **(F)** Immediate post-stenting angiogram of the left circumflex artery.

After percutaneous coronary intervention, he was treated with aspirin 100 mg once daily, clopidogrel 75 mg once daily, atorvastatin 20 mg nightly, dapagliflozin 10 mg once daily, bisoprolol 2.5 mg once daily and viliximab 2.5 mg once a day. Because the blood pressure monitoring during hospitalization suggested that the blood pressure fluctuated around 80/60 mmHg for most of the time, sacubitril valsartan was not given. His chest tightness and dyspnea improved. One month later, he was readmitted for recurrent chest tightness and dyspnea. B-type natriuretic peptide was 2,627.40 ng/L (reference 0–100 ng/L), while cardiac enzymes and blood counts were normal. Platelet aggregation testing (continuous counting method) showed ADP-induced maximal aggregation of 33.3% and arachidonic acid-induced maximal aggregation of 22.1%. Electrocardiography showed sinus rhythm (68 bpm) with complete left bundle branch block (QRS 144 ms). Echocardiography revealed a striking increase in LV size (LV end-diastolic diameter 113 mm), thinning of the anterior septum, anterior wall, inferior wall, and lateral wall from the papillary muscle level to the apex (inferior wall thickness approximately 3 mm), and an outward bulging, aneurysm-like deformity of the apical inferolateral wall with stiffness and paradoxical motion, measuring approximately 74 mm × 50 mm. Severe mitral regurgitation was present without leaflet or chordal abnormalities, consistent with secondary (functional) regurgitation (Figure 3). Bedside chest ultrasound showed massive bilateral pleural effusion.

**Figure 3:**
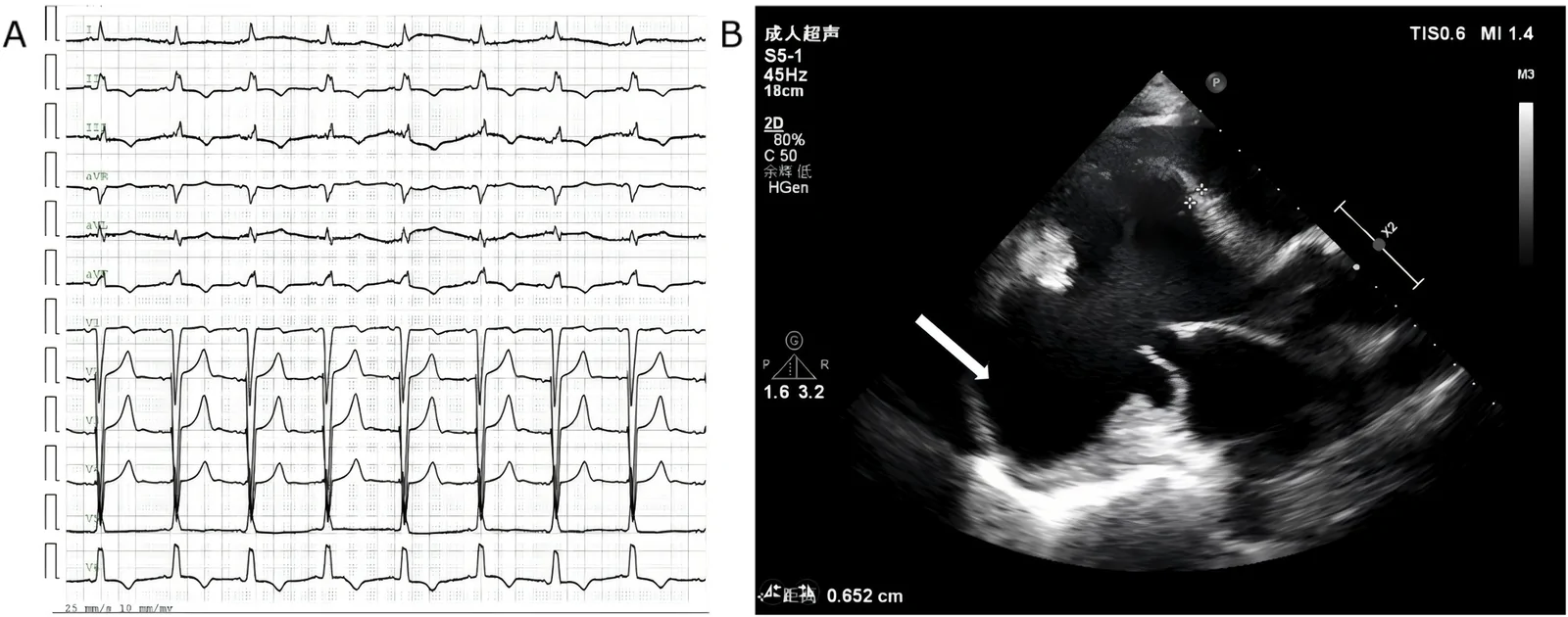
**(A)** Electrocardiogram at the second admission. Sinus rhythm, heart rate 68 bpm, QRS duration 144 ms, QTc 526 ms. **(B)** Transthoracic echocardiography; the white arrow indicates the left ventricular aneurysm(74 mm × 50 mm).

He received intensified heart failure therapy with symptomatic improvement. Repeat coronary angiography was recommended, but he and his family declined. He was subsequently transferred to Wuhan Union Hospital for advanced therapy and underwent heart transplantation for end-stage heart failure. Grossly, the explanted heart showed marked LV dilatation and a large aneurysmal segment at the inferolateral apical region. Histopathology of tissue obtained adjacent to the LV aneurysm showed features consistent with myocardial infarction accompanied by chronic inflammatory cell infiltration; residual myocytes had blurred striations and enlarged, hyperchromatic nuclei. Histopathological examination of the sampled LCX segment demonstrated atherosclerotic stenosis (Figure 4).

**Figure 4:**
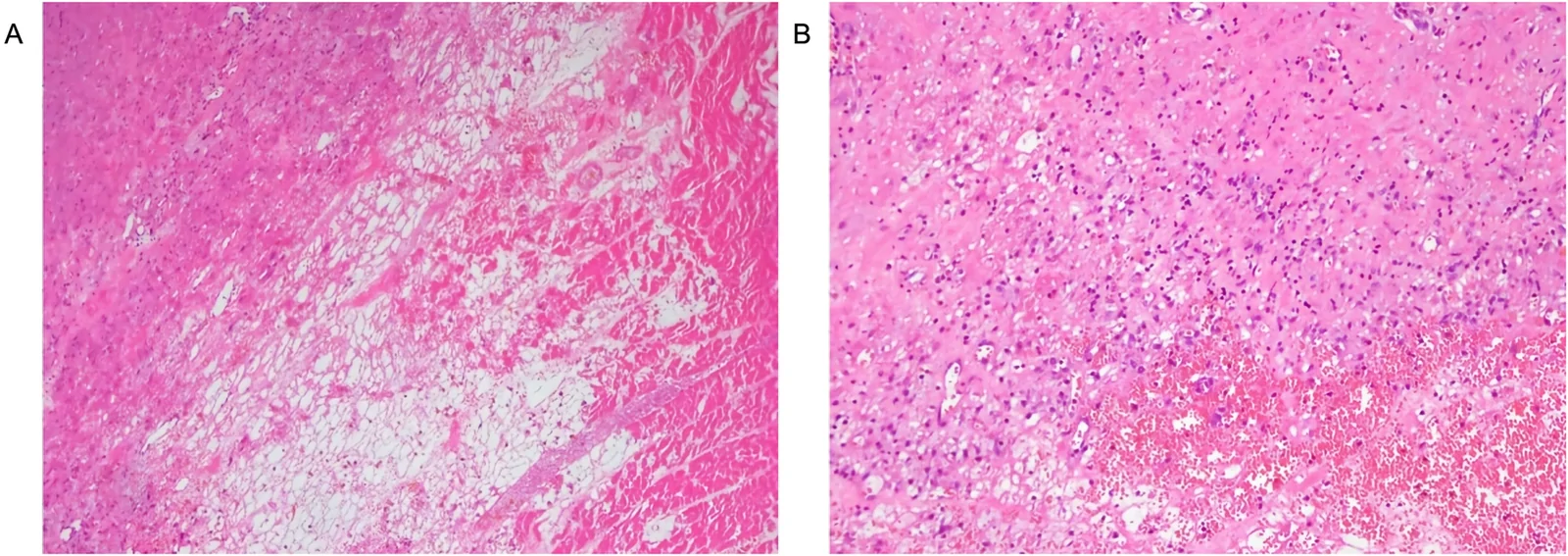
Hematoxylin and eosin–stained sections from the aneurysm specimen. **(A)** ×40. **(B)** ×100.

In summary, the patient was admitted with new-onset heart failure symptoms and moderate LV dilatation with reduced ejection fraction, underwent LCX revascularization during the index hospitalization, and then experienced rapid remodeling over the subsequent month with massive LV dilatation, development of an inferolateral apical LV aneurysm and severe functional mitral regurgitation, ultimately progressing to end-stage heart failure requiring transplantation.

## Discussion

This case highlights an uncommon but clinically important scenario: newly diagnosed DCM with rapid LV remodeling complicated by an LV aneurysm attributable to ischemic injury. LV aneurysm most commonly follows anterior transmural myocardial infarction, whereas aneurysms reported in idiopathic DCM have often been described in posterior basal regions (7). Proposed mechanisms in DCM include regional fibrosis, intraventricular conduction disturbance, and mechanical dyssynchrony, which produce heterogeneity in contraction and regional wall stress; prior work has suggested that wall stress may be higher in basal segments than in apical segments in DCM (8).

Myocardial infarction-related ventricular wall aneurysms are most commonly seen in transmural anterior wall myocardial infarction. Studies have shown (9) that left ventricular ejection fraction, left anterior descending artery lesions, and the urea nitrogen-to-albumin ratio have predictive value for left ventricular aneurysm formation in patients with acute ST-segment elevation myocardial infarction undergoing primary percutaneous coronary intervention. When the urea nitrogen-to-albumin ratio is ≥0.15, LVEF is <50%, and the culprit vessel is the left anterior descending artery, the patient's risk of developing a left ventricular aneurysm increases. In this case, the patient's urea nitrogen-to-albumin ratio was 0.17, and LVEF was significantly reduced, categorizing the patient as high-risk for post-myocardial infarction ventricular aneurysm. However, the location of the aneurysm in this patient is relatively uncommon. This may be attributed to the patient's underlying dilated cardiomyopathy, coexisting left bundle branch block-like conduction delay leading to delayed contraction of the left ventricular lateral wall, combined with myocardial infarction in the left ventricular lateral wall, resulting in wall thinning and significant local wall stress disparities, which together contributed to the formation of a left ventricular inferolateral wall aneurysm.

In the present patient, the aneurysm formed at the inferolateral apical level, a location more consistent with LCX territory injury. This was supported by the initial angiographic findings (severe LCX/OM1 stenosis with slow flow) and by post-transplant histopathology demonstrating infarction in tissue adjacent to the aneurysm and atherosclerotic LCX stenosis. The exceptionally rapid progression from moderate LV dilatation to massive LV enlargement with aneurysm formation within one month suggests that DCM-related electrical and mechanical dyssynchrony, reflected by complete left bundle branch block on readmission, may have amplified regional stress in the lateral wall—especially after infarction-related thinning—thereby accelerating aneurysm development.

The patient has coronary artery disease risk factors such as diabetes and smoking, with cardiac enlargement and reduced LVEF. It is difficult to determine whether this is caused by myocardial ischemia due to circumflex artery lesions, as there is a lack of comparative examination data. However, based on the LBBB shown in the admission ECG and the significant enlargement of the left atrium and left ventricle seen in the cardiac ultrasound, the findings tend to suggest recurrent myocardial infarction superimposed on dilated cardiomyopathy. The rationale is that if the cardiac enlargement were caused by a circumflex artery lesion, it would be unlikely to result in significant left atrial and ventricular enlargement within a short period (5 days). Moreover, CAG in this case revealed right-dominant circulation, making LBBB caused by a circumflex artery lesion improbable. In conclusion, the patient likely has underlying dilated cardiomyopathy with superimposed acute coronary events, leading to further decompensation of cardiac function.

Myocarditis was considered given the antecedent “cold,” but cardiac enzymes, cytokines, and viral myocarditis antibodies were normal during the initial admission, and the pathological findings were consistent with ischemic injury and coronary atherosclerosis, making myocarditis less likely as the primary driver. Severe mitral regurgitation was most consistent with functional regurgitation secondary to marked LV dilatation and aneurysm-related geometric distortion, as echocardiography did not show structural mitral valve disease. Clinically, this case underscores that in patients with DCM—particularly when symptoms are non-specific—coexisting ischemic injury can be overlooked. Early and, when warranted, repeat ischemic assessment should be considered when regional wall motion abnormalities, conduction changes, or rapid deterioration are observed. Recognition of this overlap may influence decisions regarding revascularization, advanced heart failure therapies, and timely referral for transplantation.

## Data Availability

The raw data supporting the conclusions of this article will be made available by the authors, without undue reservation.
